# Microarray Analyses of Inflammation Response of Human Dermal Fibroblasts to Different Strains of *Borrelia burgdorferi* Sensu Stricto

**DOI:** 10.1371/journal.pone.0040046

**Published:** 2012-06-29

**Authors:** Frédéric Schramm, Aurélie Kern, Cathy Barthel, Sophie Nadaud, Nicolas Meyer, Benoît Jaulhac, Nathalie Boulanger

**Affiliations:** 1 EA 4438, Physiopathologie et Médecine Translationnelle, Facultés de Médecine et de Pharmacie, Université de Strasbourg, Strasbourg, France; 2 INSERM UMR-S 956, UPMC Université Paris 06, Paris, France; 3 Laboratoire de Biostatistique et Informatique Médicale, Faculté de Médecine, Université de Strasbourg, Strasbourg, France; Kansas State University, United States of America

## Abstract

In Lyme borreliosis, the skin is the key site of bacterial inoculation by the infected tick, and of cutaneous manifestations, erythema migrans and acrodermatitis chronica atrophicans. We explored the role of fibroblasts, the resident cells of the dermis, in the development of the disease. Using microarray experiments, we compared the inflammation of fibroblasts induced by three strains of *Borrelia burgdorferi* sensu stricto isolated from different environments and stages of Lyme disease: N40 (tick), Pbre (erythema migrans) and 1408 (acrodermatitis chronica atrophicans). The three strains exhibited a similar profile of inflammation with strong induction of chemokines (CXCL1 and IL-8) and IL-6 cytokine mainly involved in the chemoattraction of immune cells. Molecules such as TNF-alpha and NF-κB factors, metalloproteinases (MMP-1, -3 and -12) and superoxide dismutase (SOD2), also described in inflammatory and cellular events, were up-regulated. In addition, we showed that tick salivary gland extracts induce a cytotoxic effect on fibroblasts and that OspC, essential in the transmission of *Borrelia* to the vertebrate host, was not responsible for the secretion of inflammatory molecules by fibroblasts. Tick saliva components could facilitate the early transmission of the disease to the site of injury creating a feeding pit. Later in the development of the disease, *Borrelia* would intensively multiply in the skin and further disseminate to distant organs.

## Introduction

Lyme borreliosis (LB) caused by spirochetes of the *B. burgdorferi* sl group is the most common vector-borne disease in the Northern Hemisphere. These bacteria are transmitted by the tick *Ixodes* spp. [1]. LB is a multisystemic infection that starts generally with an erythema migrans (EM) lesion at the site of the tick bite. Untreated, the infection can progress and disseminate, with inflammatory complications commonly affecting distant skin sites, joints, heart, and nervous system [2]. LB differs in clinical features based upon its geographic distribution and in relation to its pathogenic potential and/or tissue tropism [3].

The skin represents a key interface in LB since it is the target of the spirochetes at the early stage of the disease, the EM and at later stages of the disease, the borrelial lymphocytoma and a typical manifestation of late european LB, the acrodermatitis chronica atrophicans (ACA) [4], [5]. The skin constitutes a complex physical barrier [6]. The external multilayered part, the epidermis, mainly composed of keratinocytes (KCs) and Langerhans cells, is tightly connected to the dermis, in which fibroblasts are the main resident cells [7]. Dermal fibroblasts not only play an active role in synthesizing and remodeling the extracellular matrix (ECM), but also communicate with other cell types such as dermal dendritic cells, mast cells, macrophages and KCs. They also participate in tissue homeostasis, leukocyte recruitment and inflammation regulation [8]. Due to their broad and highly specialized roles in conditioning the cellular and cytokine/chemokine environment, resident sentinel fibroblasts function as part of the immune system [9].

To date, most studies of the cutaneous phase of LB have focused on the interaction of *Borrelia* with dendritic cells [10], [11], mast cells [12], and KCs [13], [14]. A few studies have investigated fibroblast responses to this disease. A recent study indicated that the interaction of *B. burgdorferi* ss with dermal fibroblasts induced the proinflammatory chemokine IL-8, along with the antimicrobial peptides defensin and cathelicidin [15]. *Borrelia* has also been shown to internalize and survive within fibroblasts [16]. Although KCs are the first cells to be injured by the tick mouthparts, biting pieces penetrate deeply into the skin [17]. Spirochetes are inoculated into the dermis, interacting with additional immune cells (dermal dendritic cells, mast cells…) and the fibroblasts. We found it therefore particularly relevant to assess how *Borrelia* infection impacts dermal fibroblasts.

In this study we investigated the role of dermal fibroblasts in skin inflammation in response to *Borrelia*. Since the inflammation could be related to the specific environments from which the strains were isolated, we tested one strain isolated from a tick and two strains isolated from different stages of the disease, potentially providing a link between spirochetal-related factors and LB outcome. Toward this end, we used specific skin cDNA microarrays to compare the global transcriptional response elicited in human dermal fibroblasts by three different strains of *B. burgdorferi* ss, isolated from an infected tick (N40) and from patients affected by EM (Pbre) or ACA (1408). Then, we investigated more precisely whether one of the major lipoproteins of *Borrelia*, OspC, which is necessary for the transmission of *Borrelia* to the vertebrate host [18], [19], could be responsible for the induction of inflammatory molecules secreted by fibroblasts. Finally, we tested the effect of tick salivary gland extracts (SGE) on *Borrelia*-induced fibroblast response.

## Results

### Fibroblasts Stimulated by *B. burgdorferi* ss N40, Pbre and 1408 Strains Secrete Inflammatory Genes

**Figure 1:**
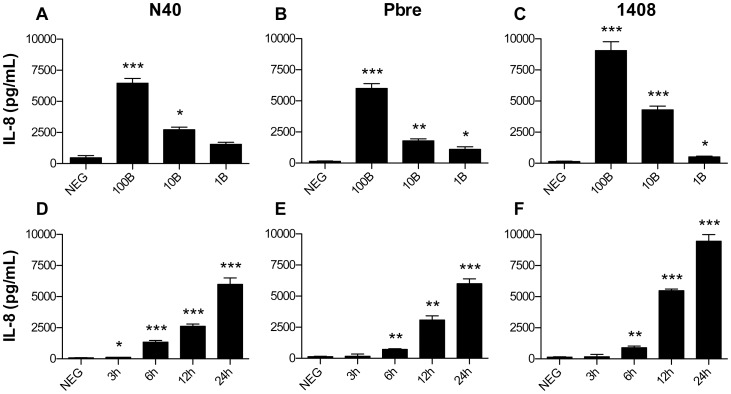
Measure of IL-8 secretion by fibroblasts co-incubated with different strains of *B. burgdorferi* ss. (A-C) IL-8 secretion of fibroblasts stimulated by different concentrations of *Borrelia* N40, Pbre, 1408 at MOI of 100∶1 (100B), 10∶1 (10B), and 1∶1 (1B) at 24 hours. (D-F) Kinetic studies of IL-8 secretions in the three strains. NEG: unstimulated fibroblasts. (A-F) Each bar shows the mean ± SDs of triplicate values and is representative of three independent experiments. ***P<0.001; **P<0.01; and *P<0.05 compared between stimulated and unstimulated cells.

*B. burgdorferi* ss N40 has been shown to induce a proinflammatory response when coincubated with human primary fibroblasts. In this response, IL-8 was induced in a dose-dependent manner [15]. To check whether *B. burgdorferi* ss N40, Pbre and 1408 behave similarly when co-incubated *in vitro* with fibroblasts, we measured IL-8 synthesis. The chemokine was secreted in a dose- and time-dependent manner, with peak secretion at 24 hours after cell stimulation (Figure 1). We then chose a 24 hours time-course for fibroblast stimulation and a multiplicity of infection (MOI) of 100∶1 for all the experiments to get the best signal.

### Global Fibroblast Transcriptional Responses to *B. burgdorferi* ss N40, Pbre and 1408

**Figure 2:**
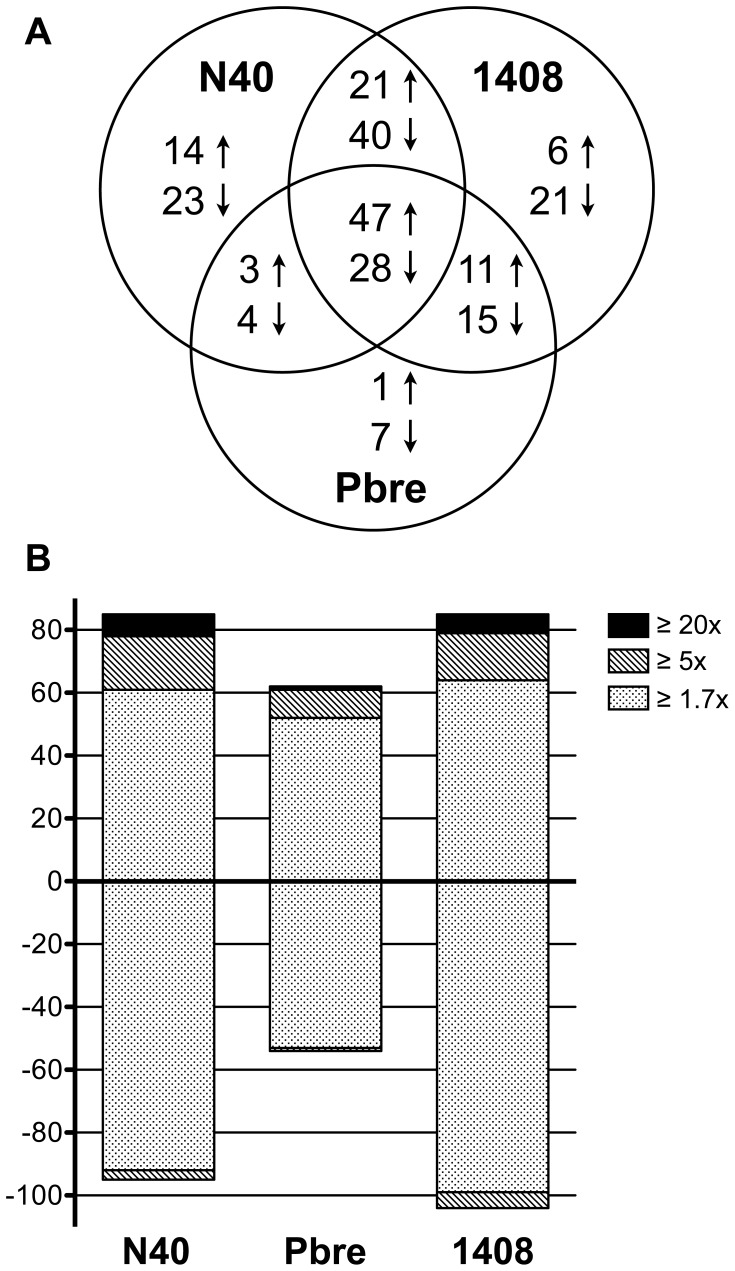
Gene expression profiles obtained from dermal fibroblasts stimulated with different strains of *B. burgdorferi* ss. (A) Venn diagram of genes significantly up-regulated (↑) or down-regulated (↓) after fibroblast stimulation with *Borrelia*, and compared with unstimulated fibroblasts. (B) Number of genes differentially expressed during fibroblast stimulation with *Borrelia*. The bars reflect the number of up-regulated genes (+) and down-regulated genes (-) for each strain. The light dotted areas correspond to gene expression changes of 1.7–5.0-fold, the grey hatched areas correspond to changes of 5.0–20.0-fold and black areas to changes ≥20.0-fold.

Two independent microarray experiments were performed for each of the three *Borrelia* strains (N40, Pbre, 1408). Statistical analysis was then performed using the 6 experiments together by comparing treated vs untreated samples. Out of 1,302 genes present on the DNA chip, 241 genes (18.5%) were differentially regulated with a fold change above 1.7 and a false discovery rate below 5% (Figure 2A). Of these 241 genes, 103 were up-regulated, 138 were down-regulated and 75 were found to be regulated by more than 1.7-fold by each of the three *Borrelia* strains (47 up- and 28 down-regulated). The majority were regulated after *Borrelia* stimulation between 1.7 and 5-fold compared to unstimulated cells for all the three strains tested (Figure 2B). This underlines that *Borrelia* has a major effect on fibroblast gene expression. The transcriptional responses induced by strain N40, Pbre and 1408 have been compared: at this point, we did not to identify relevant specific strain-related transcriptional pathway. In contrast, a notable observation was that the three *B. burgdorferi* ss strains isolated from various environments of the *Borrelia* life cycle elicited very similar transcriptional profiles in primary human dermal fibroblasts, with a core of 47 genes up-regulated in response to stimulation by all three strains.

### Up-regulated Transcriptional Responses are Largely Representative of Proinflammatory Pathways, Extracellular Matrix Synthesis and Remodeling Signals

The core of 47 up-regulated genes in response to stimulation by all three strains (Table 1), included proinflammatory genes and genes involved in ECM remodeling and synthesis. High levels of chemokines (CXCL1 and IL-8) and cytokines (IL-3, IL-6, IL-9, IL-12B, IL-13, IL-15) were induced, along with gene encoding pentraxin 3 (PTX3), a fluid-phase pattern-recognition molecule involved in the acute-phase response and innate immunity [20]. Up-regulated genes were also largely representative of intracellular signaling/regulating pathways that sustain inflammatory responses such as NF-κB transcription factors (NF-κB1, NF-κB2, Rel, RelB, IκB-α), interferon-related genes (the IFN-responsive factor IRF1, transducers of the JAK/STAT signaling cascade STAT1, STAT2 and the interferon-inducible genes OAS2 and IFIH1), and other transcription factors that could play a role in the inflammatory process (the NF-κB-induced HIF1A and JUN, component of the AP-1 transcription factor). Among genes involved in ECM remodeling, all *Borrelia* strains induced up-regulation of three matrix metalloproteinases (MMP-1, -3 and -12). Several other genes associated with cell–matrix interaction (ITGA1, the alpha subunit of the α_1_β_1_ integrin) or structural components of the ECM including microfibrils (MFAP3), collagen fibrils (COL8A1), and laminins (LAMA1) were also up-regulated by all three *Borrelia* strains. Cell activation cycle genes encoding growth factors (the KCs growth factor FGF7) and cell apoptosis-related genes encoding TNF ligand superfamily members (TNFSF10 and the B-cell activating factor TNFSF13B) and two of their receptors (TNFRSF6, TNFRSF10B), were up-regulated as well. Several other genes related to metabolism such as SOD2 were up-regulated by all three *Borrelia* strains. Among the three strains, the strain isolated from EM induced weaker inflammation than the two other strains. A large number of genes associated with intracellular metabolic functions, DNA damage repair and cell cycle control were down-regulated by one or more *Borrelia* strains (Table S1).

**Table 1:** Up-regulated genes in fibroblasts stimulated with *B. burgdorferi* in comparison to unstimulated fibroblasts.

Gene number	Annotation	N40 (tick)^1^	Pbre (EM)^1^	1408 (ACA)^1^	Description/Function
					**Inflammation**
					Chemokines
NM_001511	CXCL1	176.12	101.03	200.92	GROα, chemoattractant for neutrophils
NM_000584	IL-8	66.40	12.89	50.32	Chemoattractant for neutrophils
					Cytokines
NM_000588	IL-3	1.96	1.89	1.90	Cytokine, regulate granulocytes and monocytes-macrophages activation and proliferation
NM_000600	IL-6	39.90	6.07	13.38	Cytokine of the acute phase response
NM_000590	IL-9	1.73	2.00	1.89	Cytokine, regulates T-lymphocytes activation and proliferation
NM_002187	IL-12B	3.41	1.83	2.13	Cytokine, regulates T-lymphocytes and NK cells activation and proliferation
NM_002188	IL-13	2.47	2.68	2.44	Cytokine, regulates inflammatory and immune responses
NM_000585	IL-15	4.31	5.50	5.13	Cytokine, regulates T-lymphocytes and NK cells activation and proliferation
					Innate immunity effector
NM_002852	PTX3	9.01	3.68	13.45	Pentraxin-3 : component of the humoral arm of innate immunity
					NF-κB pathway
NM_003998	NFKB1	3.89	1.98	2.62	NF-κB p105 subunit
NM_002502	NFKB2	6.62	2.19	2.83	NF-κB p100 subunit
NM_002908	REL	4.51	2.34	3.42	C-Rel proto-oncogene protein, member of the NF-κB transcription factors
NM_006509	RELB	3.43	1.78	2.49	Member of the NF-κB transcription factors
NM_020529	IKBA	8.26	3.47	5.76	Inhibit the NF-κB transcription factor
					IFN-related pathway
NM_016817	OAS2	9.43	6.53	15.54	Oligoadenylate synthetase-2 : IFN-induced, innate immune response to viral infection
NM_022168	IFIH1	11.66	6.53	15.54	IFN-induced, alteration of RNA secondary structure
NM_002198	IRF1	6.01	2.65	6.73	Interferon regulatory factor-1 : transcription factor
NM_007315	STAT1	7.93	6.74	11.24	Signal transducer of activation-1, up-regulate genes in response to IFN type I, II or III
NM_005419	STAT2	4.83	2.29	3.68	Signal transducer of activation-2 : up-regulate genes in response to IFN type I
NM_003745	SOCS1	2.18	1.75	2.60	Suppressor of cytokine signaling-1 : negative feedback loop of the JAK/STAT pathway
NM_014011	SOCS5	2.05	1.79	1.91	Suppressor of cytokine signaling-5 : negative feedback loop of the JAK/STAT pathway
					Other transcription factors
NM_002228	JUN	2.45	2.00	3.02	Transcription factor AP-1
NM_001530	HIF1A	4.96	2.11	2.58	Hypoxia-inducible factor 1-α : NF-κB induced, role in myeloid cell-mediated inflammation
					**Extracellular matrix**
					Metalloproteinases
NM_002421	MMP-1	19.86	4.65	6.68	Matrix metalloproteinase-1 : interstitial collagenase
NM_002422	MMP-3	9.46	2.49	4.37	Matrix metalloproteinase-3 : stromelysin
NM_002426	MMP-12	14.15	2.74	5.90	Matrix metalloproteinase-12 : macrophage metalloelastase
					Components of extracellular matrix
NM_001850	COL8A1	3.10	2.06	4.58	Collagen alpha-1(VIII) chain
NM_005559	LAMA1	2.61	1.75	2.24	Laminin subunit α-1
NM_005927	MFAP3	2.29	1.91	1.72	Microfibril-associated glycoprotein-3, component of the elastin-associated microfibrils
					Cell–matrix interactions
NM_181501	ITGA1	4.51	2.15	2.39	Integrin α_1_
					**Cellular cycle**
					TNF pathways and apoptosis
NM_000043	TNFRSF6	2.37	2.04	2.41	Fas receptor, death receptor involved in apoptosis
NM_003810	TNFSF10	11.66	4.58	11.25	TRAIL, TNF-related apoptosis-inducing ligand
NM_003842	TNFRSF10B	1.86	1.82	2.38	TRAIL receptor 2, death receptor involved in apoptosis
NM_006573	TNFSF13B	19.81	8.79	25.16	BAFF, B-cell activating factor
NM_003183	ADAM17	1.91	1.75	1.83	Cleaves the membrane-bound precursor of TNF-alpha to its mature soluble form
NM_003879	CFLAR	4.95	3.44	3.30	Caspase-8 and FADD-like apoptosis regulator
					Apoptosis inhibition
NM_001165	BIRC3	4.51	4.30	6.03	Inhibitor of apoptosis protein-1
					Growth factor
NM_002009	FGF7	7.36	2.71	2.26	Fibroblast growth factor-7 : stimulates keratinocyte growth
					**Cellular metabolisms and miscellaneous**
NM_000636	SOD2	33.07	11.84	28.67	Superoxide dismutase
NM_006169	NNMT	6.85	3.39	5.15	Nicotinamide N-methyltransferase
NM_002485	NBN	3.83	3.41	2.15	Nibrin, repair of double strand breaks
NM_001539	DNAJA1	3.15	2.22	3.25	Heat-shock 40 kDa protein 4
NM_000165	GJA1	1.99	1.91	1.88	Connexin-43, component of gap junctions
NM_000345	SNCA	2.46	2.47	2.07	Alpha-synuclein, involved in membrane composition and turnover
NM_000104	CYP1B1	3.41	2.60	2.42	Belongs to the cytochrome P450 superfamily of enzymes
NM_006317	BASP1	1.91	1.71	1.82	Membrane bound protein, unknown function
-	LOC387763	22.02	6.07	14.31	Unknown function

1 For each strain, values shown correspond to the mean ratio of the duplicate measurement determined between normalized gene intensity values obtained after 24 hours of fibroblast stimulation with *B. burgdorferi* (MOI 100∶1) compared with gene intensity values from unstimulated cells.

### Validation of Selected Genes Among those Found to be Differentially Regulated by Microarray Analysis

**Figure 3:**
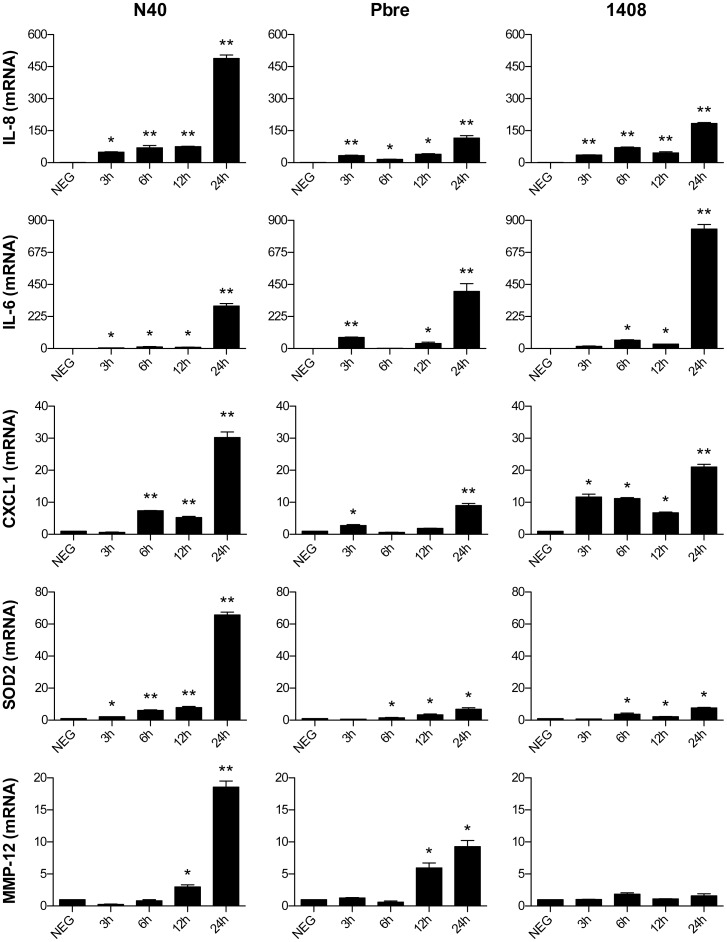
QRT-PCR analysis of mRNA expression induced by *Borrelia* in kinetic experiments with fibroblasts. The mRNA levels of IL-8, IL-6, CXCL1, SOD2 and MMP-12 were normalized to the β-actin housekeeping gene level and expressed as relative changes in gene expression compared with untreated cells (NEG). Each bar shows the mean ± SDs of triplicate values and are representative of three independent experiments. **P<0.01; and *P<0.05 compared between stimulated and unstimulated cells.

The mRNA expression of selected genes (IL-8, IL-6, CXCL1, SOD2, MMP-12) was analysed in kinetic experiments, at 3, 6, 12 and 24 hours after *Borrelia* stimulation. A similar trend in transcriptional induction was observed by quantitative reverse transcriptase polymerase chain reaction (QRT-PCR) and microarray. We confirmed strong up-regulation of the genes encoding IL-8, IL-6, CXCL1, and SOD2 for all the three *Borrelia* strains (Figure 3). We were not able to confirm the up-regulation of MMP-12 mRNA observed in the microarray by QRT-PCR for the strain 1408. The effects of *Borrelia* stimulation on these gene expressions were time-dependent, with maximal responses observed 24 hours after fibroblast stimulation. All QRT-PCR data normalized to the β-actin were further confimed by normalizing them to the expression of the RNA polymerase II, another housekeeping gene known to be stable under various stimulatory conditions [21]. Results obtained after β-actin and RNApol2 normalization were very similar to each other (data not shown).

### Effect of OspC and SGE on Fibroblast Inflammation

**Figure 4:**
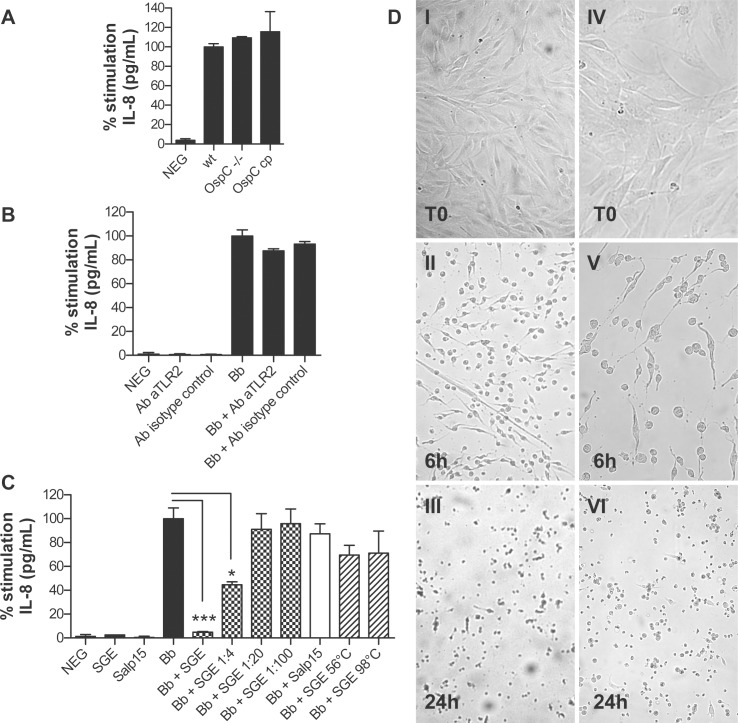
Role of OspC, *I. ricinus* salivary gland extracts (SGE) and Salp15 in *Borrelia*-induced fibroblast inflammation (A) IL-8 synthesis induced by wild-type strain 297 (wt), OspC-deficient (OspC −/−), and OspC-deficient strain 297 complemented with a plasmid carrying the ospC gene (OspC cp) in fibroblasts. (B) IL-8 synthesis in fibroblasts induced by *B. burgdorferi* ss N40 (Bb) in absence or in presence of human anti-TLR2 antibody (Ab aTLR2) or isotype control antibody (Ab isotype control). (C) IL-8 synthesis in fibroblasts coincubated with 20 µg/ml SGE alone, 30 µg/ml Salp15 alone, *B. burgdorferi* ss N40 (Bb) alone, with the combination of *Borrelia* and SGE at 20 µg/ml (Bb + SGE), 5 µg/ml (Bb + SGE 1∶4), 1 µg/ml (Bb + SGE 1∶20), and 0.2 µg/ml (Bb + SGE 1∶100), with the combination of *Borrelia* and Salp15 (Bb + Salp15), or with the combination of *Borrelia* and 20 µg/ml SGE heat-denaturated at 56°C for 1 hour (Bb + SGE 56°C), or at 98°C for 3 minutes (Bb + SGE 98°C). For (A), (B) and (C) fibroblasts were incubated with *Borrelia* at MOI of 100∶1 for 24 hours. The negative control was unstimulated cells (NEG). Each bar shows the mean ± SDs of triplicate values (expressed as % stimulation of IL-8 synthesis induced by *Borrelia* alone) and is representative of three independent experiments. ***P<0.001; and *P<0.05 compared with the corresponding stimulation induced by *Borrelia* alone. (D) Images of fibroblast cell cultures stimulated with SGE, showing SGE-induced cytotoxic effect at 6 and 24 hours (h). Images were taken at 100x (I, II and III) or at 200x magnification (IV, V and VI).

OspC is a surface lipoprotein, essential for *Borrelia* transmission to the vertebrate host. In addition to its role on KCs inflammation [14], we tested whether OspC could also be responsible for fibroblast inflammation induced by *Borrelia*. Using an OspC-deficient *Borrelia* mutant, comparable levels of IL-8 synthesis were noted 24 hours after stimulation with wild-type spirochetes, OspC-deficient *B. burgdorferi*, or OspC-deficient *B. burgdorferi* complemented with OspC (Figure 4A). These data indicate that OspC is not responsible for *Borrelia*-induced proinflammatory responses in skin fibroblasts; other surface-exposed proteins of *Borrelia* could induce that activity. To further test the contribution of *Borrelia* lipoproteins to fibroblast inflammatory response, lipoprotein signaling was blocked by anti-TLR2 antibody before *Borrelia* stimulation. The blocking effect of anti-TLR2 antibody, already tested in the interaction OspC–keratinocytes [14], only slightly (13±2%) and not significantly inhibited IL-8 secretion (Figure 4B), indicating that *Borrelia*-induced fibroblast stimulation is TLR2- independent. Tick saliva affects different cells in the skin [22] but its effect on fibroblast inflammation was never investigated in detail. Co-incubation of fibroblasts with *Borrelia* and *I. ricinus* SGE (20 µg/ml) showed a dramatic decrease of IL-8 synthesis (Figure 4C). Microscopic observation of the fibroblast cultures revealed a cytotoxic effect of SGE confirmed by cell staining with Trypan blue (data not shown). A significant morphologic change of fibroblasts was already observed at 6 hours (Figure 4D, panels II and V) leading to a mortality rate >90% 24 hours after stimulation (Figure 4D, panels III and VI). Using serial dilutions of SGE, the ability of fibroblasts to synthesize IL-8 was almost completely restored when SGE dilution reached a dilution of 1∶20 (Figure 4C), and the cytotoxic effect was reversed at the same dilution (data not shown). The decrease of *Borrelia*-induced IL-8 synthesis in presence of SGE should obviously be considered as inability of IL-8 synthesis related to SGE-induced cell death (Figures 4C and 4D). As Salp15 is a tick protein affecting various immunological processes [23], we tested whether the observed cytotoxic effect was due to this protein. Salp15 alone had no toxic effect on fibroblast cultures and did not inhibit IL-8 synthesis when coincubated with *Borrelia*. Heat-denaturation of SGE largely restored the ability of fibroblasts to synthesize IL-8 (Figure 4C) and completely abolished SGE cytotoxic effect (data not shown), indicating that SGE cytotoxic activity is linked to a proteinaceous compound present in tick saliva.

## Discussion

The skin is a major organ in the development of LB since it constitutes the inoculation site for *Borrelia* and tick saliva, and for the early and late manifestations, EM and ACA respectively [4], [5]. During and after the long-lasting blood meal of the ixodid tick, spirochetes multiply locally and interact with skin cells – dendritic cells, mast cells, fibroblasts and KCs – before migrating and reaching other tissues responsible for systemic clinical symptoms. Ticks first dilacerate the epidermis containing the KCs, then the dermis where saliva affects immune cells and the resident cells of the dermis [17]. Therefore, the immune response of the skin is essential to control the development of the disease [24]. Fibroblasts play a key role in this cutaneous immunity by cooperating with other immune cells. Fibroblasts also affect the maturation of dendritic cells [9]. Considering the tight relationships between fibroblasts, other skin cells and *Borrelia*, it was of particular interest to study the interaction fibroblasts–*Borrelia*.

We previously showed that the co-incubation fibroblasts–*Borrelia* induces antimicrobial peptides and IL-8 synthesis. Different *Borrelia*-host cell ratios were studied 100, 10 and 1 *Borrelia* for one cell. Because the MOI 100∶1 gave the strongest inflammatory response, we selected this range to study the global inflammatory response [15]. By using microarray analysis, we extended the panel of inflammatory molecules studied using different isolates of *B. burgdorferi* ss strains. Genes shown to be strongly up-regulated by microarray with all three strains were mostly related to proinflammatory signals – IL-6, IL-8 and CXCL1– that were further validated by QRT-PCR analysis and also at the protein level for IL-8 by ELISA. These molecules allow immune cell recruitment and differentiation in damaged tissues [25]. Other studies reported *Borrelia*-induced cytokine and chemokine expression (incuding IL-6, IL-8, CXCL1, CXCL9, CXCL10, CCL2, and CCL5) in primary human dermal fibroblasts [13], [26]. Müllegger *et al.,* by using QRT-PCR on skin biopsies, also found similar chemokine and cytokine induction in EM and ACA, with low but significant mRNA levels of CXCL1 and the dendritic cell chemoattractant CCL20, intermediate levels of the macrophage chemoattractant CCL2, and high levels of the T-cell-active chemokines CXCL9 and CXCL10 [27]. Skin manifestations of LB – EM and ACA – exhibit dermal infiltrate, composed predominantly of lymphocytes and histiocytes [28], [29], [30]. With regard to the potent proinflammatory response elicited by *Borrelia* in fibroblasts, dermal fibroblasts could therefore be considered as central mediators in immune cell recruitment to the skin site of *Borrelia* invasion. Their relevance in the immune response has been lately emphasized by their role on the maturation of dendritic cells [9].

MMPs are molecules important in tissue modeling. Induction of MMP synthesis by resident skin cells facilitates *Borrelia* migration from the skin to other organs. In our microarray analyses, MMP-1, -3 and -12 were found to be up-regulated by *Borrelia*. MMP-1 and MMP-3 have previously been reported in patients with Lyme arthritis [31] and in *in vitro* models of Lyme arthritis using cartilage explants and chondrocytes [32], [33]. MMP-12, involved in matrix elastin and other basement membrane component degradation [34], was up-regulated by *Borrelia* in skin fibroblasts. Interestingly, ACA represents a skin manifestation where elastic fibers are destroyed [29]. Moreover, *Borrelia* was previously found to induce MMP-12 in dendritic cells [35]. We observed a discrepancy between microarrays and QRT-PCR results for MMP-12. This lack of correlation was already described for the two techniques in similar studies, analyzing the interaction immune cells–*Borrelia* [36], [37].

*Borrelia* also induced genes related to metabolism, including SOD2, an enzyme specifically involved in oxidative burst protection. *B. burgdorferi* is known to elicit oxidative burst in immune cells [38], [39] and the role of *Borrelia*-induced reactive oxygen species in patients with EM has been postulated [40]. SOD activity has been shown to be one of the mechanism by which fibroblasts protect against oxygen reactive intermediates generated by cytokines and bacterial cell components [41]. All three *Borrelia* strains tested led to a strong up-regulated expression of SOD2 that could function as a protective mechanism by which fibroblasts counteract potential oxidative bursts elicited by *Borrelia*. We also observed up-regulation of factors involved in the IFN pathway, confirming the role of this inflammatory response to *Borrelia* [36], [37].

In our study, the three strains isolated from various environments induced a very similar inflammatory profile in fibroblasts. So, no specific strain-related pathway has been identified that could link transcriptionnal responses elicited by clinical strains 1408 (isolated from ACA), Pbre (isolated from EM) or strain N40 (isolated from a tick). Host-related factors are important, as ACA is predominantly observed in elderly patients, particularly women, and affects primarily sun-exposed acral parts of the body [42]. Spirochetal factors are also likely to play a key role in dermatoborreliosis outcome, since *B. afzelii* is the most common genospecies associated with ACA whereas *B. burgdorferi* ss and other genospecies are rarely isolated from this late clinical feature of LB [43]. However, we compared the fibroblast response of three strains of *B. afzelii* in our *in vitro* culture system (one strain isolated from an EM lesion and two strains isolated from ACA lesions) to the *B. burgdorfer*i ss strain 1408 (isolated from ACA). IL-8 release from fibroblasts co-incubated with these different strains of *B. burgdorferi* sensu lato did not differ significantly (data not shown).

A switch from OspA to OspC occurs during the migration of *Borrelia* from the midgut to the salivary glands of the tick [44]. OspC is important in the transmission to the vertebrate host [18], [19], and this lipoprotein is also described as a sensing molecule allowing *Borrelia* to migrate through the skin tissue. By inducing VEGF (Vascular endothelial growth factor), OspC may affect the vascular permeability facilitating bacterial dissemination [45]. We then tried to determine whether OspC might be responsible for part of the fibroblast inflammation. When we tested an OspC-deficient mutant [46], the inflammatory response was not modified. Moreover, the blockade of lipoprotein signaling by anti-TLR2 antibody only slightly inhibited fibroblast stimulation by *Borrelia*. These results indicate that OspC is not a major surface protein involved in *Borrelia*-elicited proinflammatory responses of fibroblasts and that other surface-exposed *Borrelia* proteins, like the integrin-binding protein P66, could elicit that role by direct interaction with fibroblasts or by interaction with the ECM components they produce [47], [48], [49]. As *Borrelia* is able to invade fibroblasts by interacting with integrins [16], and that P66 was shown to affect both endothelial and epithelial cells transcriptionnal responses [50], it could be interesting to further explore this type of interaction.

In addition to the antialarmin effect of SGE on KCs [14], we also demonstrate a lytic effect of SGE on dermal fibroblasts and that this cytotoxic effect was of proteinaceous nature and not related to Salp15. This tissue lysis induced by tick SGE could explain the feeding pit described in literature during the tick bite [51] and observed *in vivo* by intravital microscopy (Bockenstedt –personal communication). In a recent study, Hajnická *et al*. demonstrated that SGE of hard ticks displayed an inhibitory effect on cell proliferation in a mouse cell line, reduced cell adherence and induced morphologic changes in human cell lines [52]. The lytic action of *Ixodes* SGE *in vitro* on human primary fibroblasts could be linked to this effect in the days immediately following the tick bite. Tick saliva counteracts skin wound repair by its inhibitory effects on hemostasis (coagulation, platelet aggregation, and vasoconstriction), inflammation and innate immunity, thus avoiding tick rejection and allowing maintenance of tick attachment to the host during blood feeding [53]. The effect of tick saliva on the skin occurs rapidly and is strictly limited to the tick bite. We hypothesized that after the tick detaches, the saliva effect decreases and *Borrelia* can multiply intensively locally as shown in different mouse models, especially at day 7 after syringe inoculation [45], [54], [55]. This intense *Borrelia* multiplication in the skin likely corresponds to a high ratio pathogens–host cell, at a certain point during the early transmission, not too far from the one we used *in vitro* in our assay. Once the clinical manifestations appear, a few weeks to few months after the tick bite, *Borrelia* interacts with fibroblasts at different time points, first in EM, then later in ACA, inducing an inflammatory response similar to those observed in the microarray assays.

## Materials and Methods

### Spirochete Strains

Three european strains of *B. burgdorferi* ss were selected: N40 isolated from a tick, and two strains (Pbre and 1408) isolated from skin biopsies of EM and ACA respectively. *B. burgdorferi* ss 297 and its OspC-deficient relative mutant have already been described [46]. All strains were used at passage 5 to 8, cultured in BSK-H medium (Sigma, Saint Quentin Fallavier, France) at 33°C and washed before the assays.

### Tick Salivary Glands and Salp15

SGE of *I. ricinus* was prepared as described previously [14]. Absence of endotoxin was checked by the *Limulus* assay before use, and an equivalent of salivary glands of one tick (around 20 µg/ml) was used. For the assays with heat-denaturated SGE, extracts were incubated at 56°C for 1 hour, or at 98°C for 3 minutes before use. Purified Salp15 from *I. ricinus* was used at a concentration of 30 µg/ml, as described previously [14].

### Fibroblast Culture and Stimulation

Primary human dermal fibroblasts (NHDF, Promocell, Heidelberg, Germany) were maintained in FGM2 medium. To stimulate the cells, fibroblasts were used at passage 3 to 5 and seeded at 7.5×10^4^ per well in a 12-well plate. At confluence and one day before *Borrelia* activation, FGM2 medium was replaced by FGM medium without fetal calf serum. If not otherwise stated, fibroblasts were stimulated with *B. burgdorferi* spirochetes at MOI of 100∶1 for 24 hours. For the assays with tick SGE or with Salp15, spirochetes were preincubated for 30 minutes with the tick compounds at room temperature, and the preparation was then transferred onto fibroblast cells and further incubated for 24 hours. For the assays with TLR2-blocking antibody, the anti-human TLR2 antibody and its isotype control antibody (eBioscience, Ltd., United Kingdom) were used at 5 µg/ml and incubated for 30 min at room temperature on fibroblasts. *Borrelia* (at MOI of 100∶1) was then added, and the samples were further incubated for 24 hours. Before collecting stimulated or unstimulated fibroblasts in Trizol (Invitrogen, Cergy-Pontoise, France), the viability of cells was checked by Trypan blue staining.

### ELISA

IL-8 secretion was measured in supernatants of unstimulated and *Borrelia*-stimulated cells by ELISA. The protocol was based on sandwich techniques, as described by the manufacturer (R&D systems, Lille, France).

### RNA Extraction and Quantitative Real Time RT-PCR

After removal of the supernatant, fibroblasts were directly suspended in Trizol for RNA extraction according to the manufacturer’s protocol. After treatment with DNase (Ambion, Courtaboeuf, France), 2 µg of total RNA was reverse-transcribed with the Superscript II first-strand synthesis system (Invitrogen, Cergy-Pontoise, France). Semiquantitative reverse transcription PCR (QRT-PCR) was done on a LightCycler system 2.0 (Roche, Meylan, France) with specific primers (Table S2). Expression levels of all transcripts studied were normalized to housekeeping gene level and the relative changes in gene expression were compared with those of untreated cells using the 2^−ΔΔCt^ method. Two housekeeping genes were tested: β-actin and the RNA polymerase II genes [21].

### Microarray Analysis

The topic-defined PIQOR™ Skin cDNA Microarray (Miltenyi Biotec GmbH, Bergisch Gladbach, Germany) comprising 1,302 genes was used to generate gene expression profiles of Cy5-labeled unstimulated versus Cy3-labeled *Borrelia*-stimulated fibroblasts. All steps of the microarray process (including hybridization, scanning, and data analysis) were performed as described elsewhere in detail [56]. Data were based on independent duplicate measurements for each *Borrelia* strain (N40, Pbre, 1408) and inter-array normalization was performed by median normalization using BRB-ArrayTools developed by Dr. Richard Simon and the BRB-ArrayTools Development Team (http://linus.nci.nih.gov/BRB-ArrayTools.html). The normalized log-ratio values of the 6 experiments (2 experiments/strain) were analyzed with significance analysis of microarrays (SAM) [57], using the MultiExperiment Viewer (MeV, v4.0.01) software tool [58]. Regulated genes (all strains together) were selected after one-class analysis (20,000 sample permutations) with a false discovery rate (FDR) threshold of 5% and a mean change in their expression level of at least 1.7-fold. For each particular strain, each gene found regulated in the global analysis was then considered regulated if the mean fold change of the duplicate experiments for this particular strain was above 1.7. The microarray data have been deposited in the GEO repository (http://www.ncbi.nlm.nih.gov/geo/) with the record number GSE31740. Microarray experiments were performed according to the MIAME guidelines [59].

### Statistics

For ELISA and QRT-PCR, each experiment of cell stimulation with bacteria was carried out at least three times in independent experiments. Results are presented as means ± standard deviations (SDs) and were analyzed by Student’s t test. Differences in values are considered significant at p<0.05. For ELISAs, the data are the means ± SDs of triplicate values and are representative of three independent experiments. For QRT-PCR, the values are normalized to the negative control (medium alone) and shown as the fold number of the control’s value. The means ± SDs of triplicate values were compared between stimulated and unstimulated cells and are representative of three independent experiments.

## Supporting Information

Table S1 **Down-regulated genes in fibroblasts stimulated with** ***B. burgdorferi*** **in comparison to unstimulated fibroblasts.** ^1^ For each strain, values shown correspond to the mean ratio of the duplicate measurement determined between normalized gene intensity values obtained after 24 hours of fibroblast stimulation with *B. burgdorferi* (MOI 100∶1) compared with gene intensity values from unstimulated cells. Missing values are indicated with a hyphen (-). As values are expressed as ratios, a <0.58-fold downregulation correspond to a fold change <–1.7.
(PDF)

Table S2 **Primers used for the quantitative RT-PCR.**
(PDF)
