# Supplementary material for: Microarray Analyses of Inflammation Response of Human Dermal Fibroblasts to Different Strains of Borrelia burgdorferi Sensu Stricto
Source: PLoS One. 2012 Jun 29;7(6):e40046. doi: 10.1371/journal.pone.0040046 (PMC3386942; doi:10.1371/journal.pone.0040046)
Supplement: Table S1 — Down-regulated genes in fibroblasts stimulated with B. burgdorferi in comparison to unstimulated fibroblasts. 1 For each strain, values shown correspond to the mean ratio of the duplicate measurement determined between normalized gene intensity values obtained after 24 hours of fibroblast stimulation with B. burgdorferi (MOI 100∶1) compared with gene intensity values from unstimulated cells. Missing values are indicated with a hyphen (-). As values are expressed as ratios, a <0.58-fold downregulation correspond to a fold change <–1.7. (PDF) [file pone.0040046.s001.pdf]

Table S1. Down-regulated genes in fibroblasts stimulated with *B. burgdorferi* in comparison to unstimulated fibroblasts

Gene number	Annotation	N40 (tick) [1]	Pbre (EM) [1]	1408 (ACA) [1]	Description/Function
					**Cellular cycle**
					DNA repair
NM_000107	DDB2	0.44	0.76	0.47	Component of the UV-damaged DNA-binding protein complex
NM_000400	ERCC2	0.37	0.77	0.56	Involved in nucleotide excision repair of DNA
NM_000122	ERCC3	0.62	0.61	0.52	Involved in nucleotide excision repair of DNA
NM_004629	FANCG	0.10	0.58	0.30	Operate in a postreplication repair or a cell cycle checkpoint function
NM_001515	GTF2H2	0.70	0.6	0.49	Involved in nucleotide excision repair of DNA
NM_001516	GTF2H3	0.53	0.54	0.59	Involved in nucleotide excision repair of DNA
NM_002129	HMGB2	0.37	0.65	0.39	DNA double-strand breaks repair
NM_002945	MAZ	0.46	0.41	0.32	May play a role in DNA repair
NM_002412	MGMT	0.48	0.66	0.56	Repairs alkylated guanine in DNA
NM_005590	MRE11A	0.76	0.76	0.44	Role in double-strand break repair
NM_000251	MSH2	0.35	0.46	0.24	Component of the post-replicative DNA mismatch repair system
NM_032853	MUM-1	0.38	0.45	0.34	Involved in the DNA damage response pathway
NM_001048171	MUTYH	0.26	0.62	0.28	Involved in oxidative DNA damage repair
NM_002542	OGG1	0.28	0.49	0.40	DNA repair enzyme that incises DNA at 8-oxoG residues
NM_000534	PMS1	0.61	0.73	0.48	Probably involved in the repair of mismatches in DNA
NM_006502	POLH	0.33	0.76	0.40	DNA polymerase specifically involved in DNA repair
NM_007195	POLI	0.72	0.78	0.46	Error-prone DNA polymerase specifically involved in DNA repair
NM_006904	PRKDC	0.62	0.62	0.52	Acts as a molecular sensor for DNA damage
NM_000380	XPA	0.47	0.82	0.38	Involved in DNA excision repair
					Cell cycle control/Growth factors/Apoptosis
NM_001237	CCNA2	0.40	0.68	0.46	Cyclin-A2
NM_001759	CCND2	0.48	0.47	0.40	G1/S-specific cyclin-D2
NM_004354	CCNG2	0.73	0.61	0.53	Cyclin-G2
NM_004935	CDK5	0.69	0.69	0.57	Cyclin-dependent kinase 5
NM_001807	CEL	0.19	0.60	0.30	Catalyzes fat and vitamin absorption
NM_001274	CHEK1	0.52	0.55	0.30	Cell cycle arrest in response to DNA damage
NM_004879	EI24	0.44	0.65	0.57	Etoposide-induced protein 2.4 homolog
NM_004095	EIF4EBP1	0.46	0.69	0.63	Eukaryotic translation initiation factor 4E-binding protein 1
NM_001981	EPS15	0.84	0.54	0.42	Epidermal growth factor receptor substrate 15
NM_004447	EPS8	0.70	0.34	0.36	Epidermal growth factor receptor kinase substrate 8
NM_001005862	ERBB2	0.41	0.62	0.52	Receptor tyrosine-protein kinase erbB-2
NM_002010	FGF9	0.58	0.70	0.57	Fibroblast growth factor 9
NM_000557	GDF5	0.44	0.48	0.55	Growth/differentiation factor 5
NM_000598	IGFBP3	0.48	0.53	0.37	Insulin-like growth factor-binding protein 3
NM_005916	MCM7	0.39	0.59	0.40	DNA replication initiation and elongation
NM_001033053	NLRP1	0.74	0.53	0.59	Mediator of apoptosis
NM_002514	NOV	0.58	0.70	0.56	Insulin-like growth factor-binding protein 9
NM_002873	RAD17	0.41	0.55	0.38	Cell cycle checkpoint protein RAD17
NM_181471	RFC2	0.54	0.69	0.49	Replication factor C subunit 2
NM_003118	SPARC	0.88	0.49	0.55	Regulate cell growth
NM_000660	TGFB1	0.61	0.56	0.41	Transforming growth factor β1
NM_012473	TXN2	0.55	0.71	0.61	Thioredoxin-2
NM_181573	RFC4	0.22	-	0.49	Replication factor C subunit 4
NM_181578	RFC5	0.47	-	0.47	Replication factor C subunit 5
					**Morphogenesis**
NM_006735	HOXA2	0.12	0.53	0.26	Homeobox protein Hox-A2

Gene number	Annotation	N40 (tick) [1]	Pbre (EM) [1]	1408 (ACA) [1]	Description/Function
NM_002148	HOXD10	0.51	0.59	0.61	Homeobox protein Hox-D10
NM_003240	LEFTY2	0.24	0.45	0.46	Required for left-right asymmetry determination of organ systems
					**Various cellular metabolisms and functions**
NM_005689	ABCB6	0.45	0.69	0.53	Mitochondrial ATP-binding cassette sub-family B member 6
NM_000667	ADH1C	0.42	0.74	0.20	Alcohol dehydrogenase 1C
NM_000029	AGT	0.24	0.54	0.61	Component of the renin-angiotensin system
NM_001002857	ANXA2	0.54	0.77	0.56	Annexin A2
NM_000038	APC	0.69	0.45	0.36	Tumor suppressor
NM_019893	ASAH2	0.50	0.64	0.57	Neutral ceramidase
NM_016115	ASB3	0.65	0.67	0.44	Mediates ubiquitination and proteasomal degradation of proteins
NM_004656	BAP1	0.65	0.55	0.36	Ubiquitin carboxyl-terminal hydrolase BAP1
NM_001747	CAPG	0.52	0.72	0.59	Reversibly blocks the barbed ends of actin filaments
NM_001752	CAT	0.55	0.86	0.53	Catalase
NM_016174	CERCAM	0.72	0.57	0.57	Glycosyltransferase 25 family member 3
NM_001823	CKB	0.37	0.75	0.51	Creatine kinase B-type
NM_001831	CLU	0.68	0.79	0.32	Clusterin
NM_020441	CORO1B	0.58	0.52	0.54	WD repeat-containing actin-binding proteins that regulate cell motility
NM_014325	CORO1C	0.74	0.77	0.53	WD repeat-containing actin-binding proteins that regulate cell motility
NM_003389	CORO2A	0.52	0.79	0.77	WD repeat-containing actin-binding proteins that regulate cell motility
NM_000755	CRAT	0.70	0.41	0.64	Carnitine O-acetyltransferase
NM_001885	CRYAB	0.52	0.71	0.67	Heat shock protein β5
NM_001964	EGR1	0.52	0.54	0.52	Early growth response protein 1
NM_001979	EPHX2	0.32	0.61	0.34	Cytosolic epoxide hydrolase
NM_002685	EXOSC10	0.63	0.45	0.58	Exosome component 10
NM_001444	FABP5	0.41	0.89	0.59	Epidermal-type fatty acid-binding protein
NM_004104	FASN	0.62	0.52	0.47	Catalyzes the formation of long-chain fatty acids
NM_013451	FER1L3	0.75	0.59	0.40	Calcium/phospholipid-binding protein
NM_054033	FKBP1B	0.55	0.92	0.57	Peptidyl-prolyl cis-trans isomerase FKBP1B
NM_012181	FKBP38	0.75	0.52	0.38	Peptidyl-prolyl cis-trans isomerase
NM_000147	FUCA1	0.24	0.18	0.19	Alpha-L-fucosidase
NM_000402	G6PD	0.98	0.55	0.55	Glucose-6-phosphate 1-dehydrogenase
NM_002079	GOT1	0.64	0.56	0.54	Cytoplasmic aspartate aminotransferase
NM_000561	GSTM1	0.41	0.90	0.53	Glutathione S-transferase Mu 1
NM_004285	H6PD	0.68	0.50	0.42	(GDH) Oxidizes glucose-6-phosphate and glucose
NM_032495	HOPX	0.44	0.31	0.34	Homeodomain-only protein
NM_002154	HSPA4	0.67	0.61	0.34	Heat Shock Protein 70 kDa
NM_014278	HSPA4L	0.50	0.55	0.67	Heat Shock Protein 70 kDa
NM_001541	HSPB2	0.48	0.66	0.55	Heat Shock Protein β2
NM_006308	HSPB3	0.48	0.44	0.95	Heat Shock Protein 17 kDa
NM_002383	MAZ	0.42	0.34	0.34	Myc-associated zinc finger protein
NM_033316	MFI2	0.26	0.36	0.17	Involved in iron cellular uptake
NM_002413	MGST2	0.25	0.49	0.40	Microsomal glutathione S-transferase 2
NM_004689	MTA1	0.64	0.44	0.48	Component of the nucleosome-remodeling complex
NM_005967	NAB2	0.41	0.34	0.42	Transcriptional repressor for zinc finger transcription factors
NM_002513	NME3	0.56	0.81	0.60	Nucleoside diphosphate kinase 3
NM_002452	NUDT1	0.53	0.91	0.56	7,8-dihydro-8-oxoguanine triphosphatase
NM_002539	ODC1	0.34	0.86	0.43	Ornithine decarboxylase
NM_002622	PFDN1	0.75	0.35	0.60	Prefoldin subunit 1
NM_000954	PGHD	0.38	0.76	0.72	Prostaglandin-H2 D-isomerase
NM_001012973	PLAC9	0.55	0.80	0.62	Placenta-specific protein 9
NM_002691	POLD1	0.56	0.79	0.69	DNA polymerase delta catalytic subunit

Gene number	Annotation	N40 (tick) [1]	Pbre (EM) [1]	1408 (ACA) [1]	Description/Function
NM_021173	POLD4	0.56	0.60	0.60	DNA polymerase delta subunit 4
NM_006347	PPIH	0.48	0.76	0.61	Peptidyl-prolyl cis-trans isomerase H
NM_014225	PPP2R1A	0.74	0.46	0.53	Required for proper chromosome segregation
NM_002744	PRKCZ	0.47	0.39	0.36	Protein kinase C zeta type
NM_006743	RBM3	0.53	0.78	0.66	Putative RNA-binding protein 3
NM_005415	SLC20A1	0.57	0.59	0.49	Sodium-dependent phosphate transporter 1
NM_003062	SLIT3	0.51	0.60	0.50	May act as molecular guidance cue in cellular migration
NM_005563	STMN1	0.33	0.49	0.42	Involved in the regulation of the microtubule filament system
NM_005420	SULT1E1	0.48	0.80	0.47	Estrogen sulfotransferase
NM_003186	TAGLN	0.47	0.57	0.55	Involved in calcium interactions
NM_007111	TFDP1	0.97	0.53	0.56	Transcription factor Dp-1
NM_003258	TK1	0.47	0.49	0.48	Thymidine kinase
NM_003286	TOP1	0.65	0.54	0.64	DNA topoisomerase 1
NM_016292	TRAP1	0.43	0.73	0.55	Heat Shock Protein 75 kDa
NM_001071	TYMS	0.48	0.70	0.64	Thymidylate synthase
NM_002658	UPA	0.44	0.94	0.55	Urokinase-type plasminogen activator
NM_016206	VGLL3	0.35	0.80	0.40	Transcription cofactor vestigial-like protein 3
NM_006112	PPIE	0.54	0.75	0.69	Peptidyl-prolyl cis-trans isomerase E
NM_000945	PPP3R1	0.55	0.79	0.62	Calcineurin subunit B type 1
NM_001032364	GGT2	0.54	0.71	0.64	Gamma-glutamyltranspeptidase 2
NM_004542	NDUFA3	0.57	0.71	0.67	NADH dehydrogenase [ubiquinone] 1 alpha subcomplex subunit 3
NM_199173	BGLAP	0.56	-	0.60	Gamma-carboxyglutamic acid-containing protein
NM_001873	CPE	0.78	-	0.47	Carboxypeptidase E
NM_004368	CNN2	0.55	-	0.72	Calponin-2
NM_022833	FAM129B	0.68	-	0.54	Niban-like protein 1
NM_000148	FUT1	0.32	-	0.44	Galactoside 2-alpha-L-fucosyltransferase 1
NM_002284	KRT86	0.55	-	0.59	Keratin, type II cuticular Hb6
NM_002363	MAGEB1	0.38	-	0.49	Melanoma-associated antigen B1
NM_021724	NR1D1	0.71	-	0.47	Nuclear receptor subfamily 1 group D member 1
					**Extra-cellular matrix**
NM_001999	FBN2	0.64	1.43	0.51	Structural component of elastic fibers
NM_003480	MFAP5	0.86	0.60	0.37	Component of the elastin-associated microfibrils
NM_022564	MMP16	-	0.62	0.10	Matrix metalloproteinase-16
					**Cell-matrix interactions**
NM_002204	ITGA3	0.33	0.28	0.47	Integrin α3
NM_000885	ITGA4	0.53	0.65	0.46	Integrin α4
NM_000210	ITGA6	0.51	0.71	0.55	Integrin α6
NM_001937	DPT	0.65	0.59	0.53	Dermatopontin
NM_000425	L1CAM	0.60	0.79	0.52	Cell adhesion molecule
NM_003573	LTBP4	0.56	0.44	0.43	Latent-transforming growth factor beta-binding protein 4
					**Cell architecture**
NM_001615	ACTG2	0.56	0.55	0.56	Alpha-actin-3
NM_014756	CKAP5	0.68	0.52	0.58	Cytoskeleton-associated protein 5
NM_000428	LTBP2	0.55	0.55	0.12	Role in elastic-fiber architectural organization and/or assembly
NM_018718	TSGA14	0.39	0.63	0.52	Centrosomal protein of 41 kDa
NM_006009	TUBA1B	0.49	0.86	0.72	Tubulin alpha-1B chain, component of microtubules
NM_178014	TUBB	0.93	0.52	0.65	Tubulin beta-5 chain, component of microtubules

[1]For each strain, values shown correspond to the mean ratio of the duplicate measurement determined between normalized gene intensity values obtained after 24 hours of fibroblast stimulation with *B. burgdorferi* (MOI 100:1) compared with gene intensity values from unstimulated cells. Missing values are indicated with a hyphen (-). As values are expressed as ratios, a <0.58-fold downregulation correspond to a fold change <–1.7.
