# Supplementary material for: Microarray Analyses of Inflammation Response of Human Dermal Fibroblasts to Different Strains of Borrelia burgdorferi Sensu Stricto
Source: PLoS One. 2012 Jun 29;7(6):e40046. doi: 10.1371/journal.pone.0040046 (PMC3386942; doi:10.1371/journal.pone.0040046)
Supplement: Table S2 — Primers used for the quantitative RT-PCR. (PDF) [file pone.0040046.s002.pdf]

Table S2. Primers used for the quantitative RT-PCR

Primer type		Sequence	Source or reference
Actin	Forward	5'- CCA ACC GCG AGA AGA TGA CC -3'	Designed in this study
	Reverse	5'- GAT CTT CAT GAG GTA GTC AGT -3'	
RNApol2	Forward	5'- GCA CCA CGT CCA ATG ACA T -3'	Radonic, 2004
	Reverse	5'- GTG CGG CTG CTT CCA TAA -3'	
IL-8	Forward	5'- TCT GCA GCT CTG TGT GAA GGT GCA GTT -3'	Marchal, 2011
	Reverse	5'- AAC CCT CTG CAC CCA GTT TTC CTT -3'	
IL-6	Forward	5'- CCA GAA CAG ATT TGA GAG -3'	Designed in this study
	Reverse	5'- CTA CAT TTG CCG AAG AGC -3'	
CXCL1	Forward	F : 5'- GTC ACT GTT CAG CAT CTT TTC G -3'	Designed in this study
	Reverse	R : 5'- CTG CAT CCC CCA TAG TTA AGA A -3'	
MMP12	Forward	F : 5'- TGG CAT TCA GTC CCT GTA TGG AGA -3'	Designed in this study
	Reverse	R : 5'- TCC CAC GGT AGT GAC AGC ATC AA -3'	
SOD2	Forward	F : 5'- TCG TGG CTG TGG TGG CTT CG -3'	Designed in this study
	Reverse	R : 5'- CCT GCT GGT GCC GCA CAC T -3'	
